# Acceptability of the American Board of Internal Medicine medical professionalism framework in the United Arab Emirates

**DOI:** 10.5116/ijme.6023.b4ea

**Published:** 2021-02-26

**Authors:** Halah Ibrahim, Satish Nair, Sawsan Abdel-Razig

**Affiliations:** 1 Department of Medicine, Sheikh Khalifa Medical City, Abu Dhabi, UAE; 2 Department of Academic Affairs, Tawam Hospital Johns Hopkins Medicine Affiliate, Al Ain, UAE; 3 Department of Medicine, Cleveland Clinic Abu Dhabi, Abu Dhabi, UAE

**Keywords:** Professionalism, Residency, Competency, International medical education, Middle East

## To the Editor

Though medical experts worldwide agree on the importance of inculcating professionalism into medical training, there is no consensus on how to do so.^1^ This is particularly challenging in the international medical community, where cultural, religious and social factors play a significant role in how professionalism is defined.^1^ A major challenge when considering professionalism in the global arena is the fact that most expert definitions and assessment tools have been developed in Europe and North America. The Physician Charter on medical professionalism was most notable, which was developed in 2002 through the collaborative effort of American and European Internal Medicine Boards.^2^ Within months of its creation, the charter was adopted by over 90 medical societies and translated into ten different languages.^3^ The American Board of Internal Medicine (ABIM) has since proposed six domains to define medical professionalism, including altruism, accountability, excellence, duty, honor and integrity, and respect for others.^4^ Educational scholars, however, warn of this Westernization of medical education as a new wave of “imperialism” in which Western pedagogy is exported globally without deference to local values and perspectives.^5^ Within the past decade, educators in China,^6^ Iran^7^ and the Arabian countries^8^^,^^9^ have attempted to define professionalism within their respective social and cultural contexts, with several differences. Yet, in many of these studies, the authors acknowledge constructs related to this competency that persist in differing cultural settings. Recent literature on the applicability of global definitions of professionalism leaves one to consider whether certain elements of professionalism are universally acceptable.^10^ This is particularly important in the global arena, where standardized professionalism curricula may not exist for medical training programs. These universally accepted constructs might represent essential competencies required of a global physician workforce required to meet the needs of an increasingly diverse patient population.^1^

In the United Arab Emirates (UAE), as in several countries worldwide, residency programs are currently adopting Western defined competencies, against which students are taught and assessed, often without the existence of culturally or socially relevant curricula or assessment methods in place.^11^ Amongst these competencies, professionalism poses a particular challenge as it represents beliefs, attitudes, and values defined by social constructs, within the cultural context of each society.^12^ The purpose of this study was to measure the acceptability of the ABIM domains of medical professionalism to UAE residents and faculty. A secondary goal was to observe potential differences between UAE residents, who are predominantly locally-trained UAE nationals, and the multicultural and internationally trained faculty that teach them.

We conducted a cross-sectional survey, using the Penn State College of Medicine Questionnaire,^13^ of residents and teaching faculty from Internal Medicine residency training programs in three large academic medical centers in the UAE, with a comparative group of residents and faculty from a large Internal Medicine residency program in an academic medical center in the United States (US). The questionnaire measures attitudes towards professionalism in medical education and is based on the six ABIM elements of professionalism. It includes six domains with six items in each domain, and survey respondents rate the extent to which each statement is reflected in their definition of professionalism.

Study participants found all six domains of professionalism to be important to their personal definition of medical professionalism. Specifically, the order of ascending importance was duty, excellence, honor/integrity, altruism, accountability, and respect. No significant differences were found between the US and UAE residents for all domains of professionalism. Amongst faculty, UAE faculty ranked altruism higher than US faculty. When comparing all residents to all faculty, no significant differences were found for all domains of professionalism. There were no significant gender differences noted.

In our study, the six ABIM domains of medical professionalism were all considered important to residents and faculty practicing in significantly different cultural contexts. The predefined ABIM domains considered important in Western definitions of professionalism were equally and at times more, important to UAE residents and faculty. In addition, there were no significant differences in the definitions of medical professionalism between the locally trained UAE residents and their multicultural, internationally trained teaching faculty.  The emphasis of UAE physicians on the domain of altruism may reflect the prevalent belief in the Middle East that physicians have a spiritual duty to serve patient interest above self-interest.^9^

Our study adds to medical professionalism by showing that certain qualities of medical professionalism may indeed be universal. The primary lesson learned was the importance of utilizing the appropriate instrument when studying social constructs across cultures. The instrument needed to describe attributes related to the underlying domains, rather than providing explicit definitions of those domains. Additionally, multiple descriptions were used to characterize each domain. This was important as it ensured that participant responses were specific to the attitudes and behaviors that reflected their personal definition of medical professionalism. Our findings are similar to other studies that have explored the impact of culture on the conceptualization of professionalism and found many areas of commonality and overlap. A comparative analysis of the Chinese Medical Doctor Declaration with the US-based Physician Charter on Medical Professionalism found significant overlap between the two documents.^14^ Both charters focus on the primacy of patient welfare and emphasize physician integrity and social justice.^14^ Similarly, Ho and colleagues^15^ compared constructs of medical professionalism between educational stakeholders in Taiwan and mainland China and found several areas of congruence, with both frameworks valuing clinical competence and communication skills, ethics, humanism and excellence. The authors noted that these constructs also serve as the building blocks of Western frameworks of medical professionalism.^15^ Further, in a narrative overview of three non-Western (Chinese, Arabian, Japanese) frameworks of medical professionalism, many of the attributes were not only comparable amongst the three models, but also held many similarities with Western definitions of professionalism.^15^ Concepts, such as altruism, respect and teamwork, formed the foundation for all the frameworks reviewed. These findings suggest a universality of many attributes of medical professionalism and should be reassuring to non-Western countries and educational systems that have already incorporated Western definitions of professionalism into their curricula, or who are currently developing curricula and may not have the researchers or resources necessary to develop a local or a culturally contextual definition of medical professionalism.

Our results should be viewed in light of some limitations. First, the study design was limited to identifying commonalities in definition but did not identify additional constructs that may be specific to the UAE or the Middle East. Also, only Internal Medicine residents and faculty were included. This discipline was selected because it represents one of the larger residency programs in the country and educational leaders in the department are actively working to create professionalism curricula.

This study of medical professionalism in the UAE confirms that despite social and cultural differences, the ABIM domains considered important in Western definitions of medical professionalism were also important to UAE residents and faculty. There may be universal elements of medical professionalism that can serve as a framework for the development of professionalism curricula and assessment tools in international medical schools and residency programs. Additional larger scale and multi-institutional studies are needed and should include other important stakeholders, including patients, nurses, allied health professionals and public health specialists. Future studies should assess the impact of these curricula on resident performance and patient experience.

### Conflict of Interest

The authors declare that they have no conflict of interest.
